# Operative Dentistry

**Published:** 1890-09

**Authors:** Geo. T. Thuerer


### Operative Dentistry.

BY GEO. T. THUERER, D.D.S.

1. The teeth (enamel dentine) are subject to many defects during their growth and development.

They are liable to *atrophy*, which is due to the softening of a spot or spots becoming white and opaque and is due to a lack of nutrition. The spots vary in size and position and are generally confined to the enamel. They also vary in density, may be very soft and when an excavator is applied may be easily crumbled away, and again, the density may be nearly as great as the surrounding enamel.

*Denuding*—Is a stripping or divesting the tooth of its enamel. This affection usually attacks the anterior teeth and may even extend to the bicuspids and molars. It usually takes place upon the labial surface and may vary all the way from small pits to entire loss of enamel on labial surface. The enamel may be notched at the cutting edges of the teeth and again it may be all shriveled up, especially does it occur to the 6-year molars and sometimes to incisors. It may occur to any of the teeth.

*Erosion or Chemical Abrasion.*—This affection is one that is not well understood and most generally attacks the anterior surface and is characterized by transverse grooves varying in depth and number, most numerous upon incisors, and in chemical abrasion the cutting edges are worn away gradually, commencing with the centrals and extending backward to bicuspids and molars and when mouth or teeth are closed there is an elliptical opening.

Dentine oftimes is softer than normal and in these decay rapidly progresses. Mechanical abrasion, where teeth strike upon each other, cutting edges of incisors, and are worn away.

2. *Atrophy*—Is due to a lack of nutrition. Denuding; cause is not definitely known, but is supposed to be due to a low vitality of the tooth or tooth pulp. A notched condition is characteristic of syphilis and the pitting or shrivelling to some constitutional defect or any disease from which the body was suffering, during the formation of the dental tissues, consequently showing its effect.

*Erosion or Chemical Abrasion.*—Due to a chemical action; not much is known of this affection. Mechanical abrasion, when cutting edges of the anterior teeth strike upon each other and are worn away. Softened dentine due to lowered vitality of the tooth during its formative period.

3. *Pulpitis.*—An inflammation of the pulp from exposure or any other exciting cause.

Hypertrophy or fungoid tumor is by the increased activity of the pulp causing an increased growth which projects out through an orifice of exposure.

Pulp stones or nodules are points in the pulp that become ossified and they vary in number from 1 to even 50 have been found.

In pulpitis remove all irritants and apply something to allay the irritation, as oil of cloves, carbolic acid with either sulphate or acetate of morphia. In fungoid tumor excise the growth at the orifice of exposure, cauterize with nitric acid or carbolic acid, but generally the pulp dies. In pulp nodules, if there is a single one near the orifice of exposure, extract it; after hemorrhage treat as for an inflammation, but when they are numerous and scattered the pain is considerable and you have to remove pulp and all.

4. The successional steps in formation of acute alveolar abscess. First, you have an irritant that produces irritation which causes a determination of blood to the part and a consequent disturbance of the circulation and stasis. As there is a determination of blood to the part there is dilatation of the blood vessels on account of the increased pressure and then there is swelling and exudation into the surrounding structures. This ends in resolution and chronic inflammation; or may be immediately followed by suppuration and destruction of tissue.

THE PRINCIPLES OF TREATMENT.—First, remove the irritant and diminish the circulation in part, then, if there is suppuration, establish free drainage and cleanliness. When suppuration has taken place watch that it does not discharge upon the outside of the face. After the establishment of free drainage, apply antiseptic counter-irritants. See that the system is in good condition. If much pain accompanies this, as it usually does, anodynes are indicated.

5 *Acute Abscess*—Comes from an irritation; there is heat, pain, redness and swelling; heat about the part, swelling very great, pain is acute, throbbing and lancinating, is of a bright red color on account of excessive amount of arterial blood in the part and is generally accompanied by fever with high temperature and rapid pulse, and has no fistulous opening at first but developes one.

*Chronic Abscess*—Is usually the termination of an acute abscess; is not accompanied by an abnormal amount of arterial blood in the part, abnormal swelling or redness, but instead, is of a venous color, is accompanied by a fistulous opening and does not give much pain until the patient takes a cold which settles in part causing increased action.

6. When inflammation is set up in any part there is increased action in the circulation and by this there is in the capillaries of the part a pressure abnormal. At first, the white corpuscles cling to the sides of the blood vessels thus producing a layer that does not move and as this keeps on it lessens the calibre of the channel of circulation, then the white corpuscles work their way out of the capillaries by a sort of amœboid movement which causes infiltration of surrounding parts and the white corpuscles are sometimes, though in small amounts and rarely followed by the red corpuscles. This, with the increased arterial pressure, cause the swelling and pressure upon the nerves, and the pain. From this it goes on to ulceration in the superficial structures and abscess in the deep structures.

TREATMENT—Varies with the cause upon which it depends. Remove the irritant, divert blood from part, give part rest, if the patient is debilitated build him up; should take proper hygienic care of himself.

7. In the first place, ascertain whether it is a healthy tooth that you have to deal with. If it is an approximal cavity you obtain separation either by use of disk, or by previous separation by introducing fibres of cotton, or by use of separators. If a large cavity, obtain an orifice of exposure large enough so that you can thoroughly do the excavating and introduce your filling. Avoid large undercuts, then remove the superficial decay. Now apply the rubber dam to obtain dryness, and if properly done is very valuable. Now you proceed to dry, excavate the decay, strokes of excavator should be handled with a determined (sharp excavators) stroke away from pulp. After removing all decay and softened dentine and then see (should be done first) that you have good strong borders of enamel supported by dentine. Borders should be made with sharp instruments and should be clean cut and well defined. Now obtain a retaining form by having the orifice smaller than the bottom; this is often true of crown cavities but in approximal cavities make slight undercuts. These are of such a great variety that it all depends upon the skill of the operator. Should the cavity be very deep or come close to the pulp a non-conducting material should be applied to prevent thermal changes from irritating the pulp. Where you apply a non-conductor do this first then obtain the retaining form. Especial attention should be given to the cervical border as this is the most vulnerable point of breaking down. Now polish the edges of the cavity, then proceed with the introduction of the gold. Commence at the cervical border and build toward the orifice, always keeping the edges or borders of the filling higher than the centre. Thorough condensation is very essential, therefore the gold foil should not be used in too large pieces. After the filling has been introduced a thorough finish with removal of all overhanging edges is very important for the future preservation of the filling.

8. *Hypertrophy, Recession and Tumid Gums.*—Hypertrophy or the overgrowth of the gums on account of an overfed condition. The tissue resembles the true gum, and has all of its characteristics.

Tumid gums is caused by ptyalism and accumulations upon the teeth thus causing the gums to become swollen and lose their attachment to the teeth. They bleed very easily and are very tender, and are of a dark purple color on account of increased venous blood in parts.

Recession of gums occurs in some cases where it is hard to attribute any cause or to find any, but is usually some accretion upon the teeth which causes its absorption and recession. It also occurs were decay finds place at the necks of the teeth.

